# Quantifying the number of deaths among Aboriginal and Torres Strait Islander cancer patients that could be avoided by removing survival inequalities, Australia 2005–2016

**DOI:** 10.1371/journal.pone.0273244

**Published:** 2022-08-26

**Authors:** Paramita Dasgupta, Gail Garvey, Peter D. Baade

**Affiliations:** 1 Cancer Research Centre, Cancer Council Queensland, Brisbane, Queensland, Australia; 2 School of Public Health, Faculty of Medicine, University of Queensland, Brisbane, Queensland, Australia; 3 School of Mathematical Sciences, Queensland University of Technology, Gardens Point, Brisbane, Queensland, Australia; 4 Menzies Health Institute, Griffith University, Gold Coast Campus, Southport, Queensland, Australia; The University of Sydney, AUSTRALIA

## Abstract

**Background:**

While Aboriginal and Torres Strait Islander peoples have poorer cancer survival than other Australians, absolute measures of survival disparities are lacking. This study quantified crude probabilities of deaths from cancer and other causes and estimated the number of avoidable deaths for Aboriginal and Torres Strait Islanders if these survival disparities were removed.

**Methods:**

Flexible parametric relative survival models were used to estimate reported measures for a population-based cohort of 709,239 Australians (12,830 Aboriginal and Torres Strait Islander peoples), 2005–2016.

**Results:**

Among Aboriginal and Torres Strait Islander peoples, the 5-year crude probability of cancer death was 0.44, while it was 0.07 for other causes of death. These probabilities were 0.07 and 0.03 higher than among other Australians, respectively. Magnitude of these disparities varied by cancer type and ranged for cancer deaths from <0.05 for pancreatic, prostate and uterine cancers to 0.20 for cervical and head and neck cancers. Values for disparity in other causes of death were generally lower. Among an average cohort of Aboriginal and Torres Strait Islander peoples diagnosed per year over the most recent five-year diagnosis period (2012–2016, n = 1,269), approximately 133 deaths within 5 years of diagnosis were potentially avoidable if they had the same overall survival as other Australians, with 94 of these deaths due to cancer. The total number of avoided deaths over the entire study period (2005–2016) was 1,348, with 947 of these deaths due to cancer.

**Conclusions:**

Study findings suggest the need to reduce the prevalence of risk factors prevalence, increase screening participation, and improve early detection, diagnosis and treatment rates to achieve more equitable outcomes for a range of cancer types. Reported measures provide unique insights into the impact of a cancer diagnosis among Aboriginal and Torres Strait Islander peoples from a different perspective to standard relative survival measures.

## Introduction

Aboriginal and Torres Strait Islanders, the First Nations peoples of Australia, have poorer survival following a cancer diagnosis than other Australians [1–4]. Standard net survival estimates, such as relative or cause-specific survival, can be hard to interpret and, by ignoring deaths from other (non-cancer) causes, do not provide a tangible measure of the real-world impact of cancer [5].

There is growing interest in use of alternative survival measures to obtain informative and meaningful statistics that may be of interest to patients, clinicians, and policy makers [5, 6]. For example, by recognising that cancer patients may die from causes other than the cancer itself, crude survival quantifies the real-world probabilities of cancer death and other causes of death after a cancer diagnosis [5–8]. A related measure uses these estimated probabilities to quantify the absolute number of deaths that would be avoided at a given timepoint following a cancer diagnosis if one group had the same survival experience as another group [9]. The `avoided deaths’ measure can provide insights into the absolute impact of temporal, geographical or socioeconomic differences in survival across population groups [9–11].

Aboriginal and Torres Strait Islander peoples are known to have a higher risk of mortality due to non-cancer causes than other Australians [12]. However, to the best of our knowledge there are few published estimates of survival that incorporate these other causes of death among Aboriginal and Torres Strait Islander cancer patients. One previous Northern Territory (NT) study that reported how crude probabilities of death for cancer patients varied by Aboriginal and Torres Strait status was limited in geographical coverage, number of cancer types and based on a now-dated cohort (1991–2011) [13].

Here we report crude probabilities of death from cancer and other causes by Aboriginal and Torres Strait Islander status for a contemporary population-based cohort covering a large proportion of the Australian population, extracted from cancer registries with high quality Aboriginal and Torres Strait Islander identification. The impact of survival differences is further quantified by estimating the number of deaths within five years of receiving a cancer diagnosis that could have been avoided if Aboriginal and Torres Strait Islander peoples had the same cancer survival as other Australians.

## Methods

Ethical approval was obtained from the Aboriginal Health and Medical Research Council Ethics Committee (1256/17), New South Wales (NSW) Population and Health Services Research Ethics Committee (2017/HRE0204) and the Northern Territory (NT) Department of Health and Menzies School of Health Human Research Ethics Committee (2016–2689).

Data custodian approval was obtained from four cancer registries (Queensland, Western Australia, NT, NSW) that have high quality Aboriginal and Torres Strait Islander identification [14] to access de-identified data from the Australian Cancer Database [15]. Notification of all invasive cancers (except keratinocyte cancers) to these registries is a legal requirement across Australia. Collectively, these four jurisdictions cover around 84% of Australia’s Aboriginal and Torres Strait Islander population [16].

### Cohort

Data was obtained for all cancer patients (n = 843,905) diagnosed from 2005 to 2016 (up to 2015 for NT). Vital status was based on routine annual linkage of Australian National Death Index, with follow-up of all cases to 31 December 2016. The cohort was restricted to those aged 15–84 years at diagnosis (n = 767,718), with those aged 14 and under excluded due to their unique cancer classification [17] while older Australians were excluded to match the upper age limit in published Aboriginal and Torres Strait Islander life tables [18]. Cases identified at death (4,451, 0.5%), with negative survival times (1,771, 0.2%) or with unknown Aboriginal and Torres Strait Islander status (n = 52,257, 6.2%) were also excluded from the final cohort of 709,239 persons. For each cancer type, only the first primary was considered.

Analyses are reported for 16 leading individual cancer types, all other cancers and all cancers combined.

### Life tables

Published year-specific life tables for the Aboriginal and Torres Strait Islander population, stratified by age, sex and state [18] were used for all calendar years except 2008, 2009, 2013 and 2014. The life tables for these missing years were estimated using linear interpolation. Corresponding life tables for all Australians [19] were used for other Australians who comprise around 97% of the total population [16].

### Survival

Survival was measured in days from the date of diagnosis to one of death or the study endpoint (31 December 2016), whichever was earliest. Cases alive at the end of follow-up were censored.

Survival was analysed in the relative survival setting, that is as the ratio of the observed survival for the cancer cohort and the expected survival for the general population [5].

### Outcome measures

Two key outcome measures are reported, the first being the crude probability of death which partitions the all-cause probability of death (1 minus all-cause survival) into the probability of death for cancer and other causes in the relative survival setting [8]. Estimates were standardised to the covariate distribution of the Aboriginal and Torres Strait Islander cohort to allow fair comparisons across the two population groups. The second measure builds on the first and reports the average number of ‘avoidable deaths’ per year if Aboriginal and Torres Strait Islander cancer patients had the same survival as other Australians. Both measures were estimated at five years since cancer diagnosis, similar to previous studies [9, 11, 20].

### Statistical analysis

#### Modelling

For each cancer type we fitted flexible parametric relative survival models on the log cumulative excess hazard scale that used restricted cubic splines to estimate the baseline cumulative excess hazard [21]. Included covariates were age at diagnosis, sex, state/territory, and Aboriginal and Torres Strait Islander status. Age was modelled as a continuous variable using restricted cubic splines with four degrees of freedom (df). Models for all cancers combined, all other cancers and head and neck cancers also included a four-categorical variable for broad cancer type based on five-year relative survival estimates for Australia (0–24%, 25–49%, 50–74%, 75–100%) along with interactions between broad cancer type and age or sex to account for variations in case mix across diagnosis years and population sub-groups.

All variables were included as time-dependent effects based on likelihood ratio tests. However, final models did not include two-way interactions between Aboriginal and Torres Strait Islander status and other covariates (likelihood ratio tests).

Depending on cancer type, 2–5 df (1–4 internal ‘knots’) were used for the baseline and 2–6 df (1–5 internal ‘knots’) for time-varying effects. The selection of the number of knots in each instance was based on minimizing the Bayesian Information Criterion. Knots were placed at the default locations for flexible parametric relative survival models [21].

The expected mortality rate was calculated from published life tables stratified by calendar year, age, sex, state/territory and Aboriginal and Torres Strait Islander status [18, 19]. Five-year excess mortality rates were calculated separately for Aboriginal and Torres Strait Islander and other Australian cancer cohorts.

#### Crude probabilities of death

Standardised crude probabilities of death due to cancer and other causes within five years of diagnosis by Aboriginal and Torres Strait Islander status, along with the differences between these probabilities across the two population groups [20, 22], were estimated by transforming the fitted model parameters [8]. The standardised probability of dying from any cause (1- all cause survival) within five years of diagnosis), required for the avoidable death calculations, was also estimated.

The Stata postestimation *standsurv* package [23] was used to estimate the crude probability of death due to cancer and other causes [20, 22] over the entire study period (2005–2016) at five years since diagnosis with associated 95% confidence intervals (CI) through transformation of the fitted model parameters [8]. This approach allowed estimation of the crude probabilities at specific time points averaged over the corresponding predicted values over every individual in our cohort, based on their observed covariate values in the dataset. Restricting the covariate distribution over multiple confounders (in this case age, sex, state/territory) to that for Aboriginal and Torres Strait Islander peoples, enabled comparisons that solely focussed on the disparities between the two groups of interest: Aboriginal and Torres Strait Islanders and other Australians. For both cancers and other causes, corresponding probabilities of deaths for Aboriginal and Torres Strait Islanders and other Australians were comparable because they were both averaged over the exact same distribution of confounders (i.e. for Aboriginal and Torres Strait Islanders). The differences in the model-based crude probability of death from cancer and other causes across the two population groups were also estimated using the `contrasts’ option of *standsurv* [20, 22]. All CI were calculated using delta method.

In addition, the crude probability of death from cancer and other causes and the difference between these probabilities by Aboriginal and Torres Strait Islander status across the two population groups by two six-year time periods (2005–2010, 2011–2016) are presented only for all cancers combined, since many of the cancer type-specific models for the shorter diagnostic periods did not converge.

#### All-cause probability of death

For each cancer type, the standardised total (all-cause) probability of death (1-all-cause survival) with associated 95% confidence intervals at five years since diagnosis was also estimated from fitted models [20, 22] using failure option of *standsurv* package [23]. Estimates were standardised to the covariate distribution of the Aboriginal and Torres Strait Islander cohort.

#### Avoidable deaths

These calculations were based on previously published methods for determining avoidable death in the framework of flexible parametric relative survival models [9, 20]. Note that all estimates were based on survival measures standardized to the covariate distribution for Aboriginal and Torres Strait Islander people diagnosed with cancer.

The calculation of avoidable (all cause) deaths required several steps. First, the predicted number of deaths among Aboriginal and Torres Strait Islander peoples diagnosed with cancer was calculated by multiplying the number of people diagnosed in a typical calendar year (2012–2016), NumDiag_FN_ by the probability of death due to any cause t years after diagnosis, ProbDth_All_FN_ (*t*).


$${PredDth}_{All\_FN}(t)={ProbDth}_{All\_FN}(t)\times {NumDiag}_{FN}$$ (1)

Second, the expected number of deaths was calculated by taking the hypothetical scenario in which Aboriginal and Torres Strait Islander peoples diagnosed with cancer had the same relative survival as experienced by other Australians, resulting in an adjusted version of the probability of death (ProbDth_ALL_FN_ADJUST_ (*t*)). Under this assumption, the expected number of deaths among Aboriginal and Torres Strait Islander cancer patients was calculated:

$${ExpDth}_{All\_FN}(t)={ProbDth}_{All\_FN\_ADJUST}(t)\times {NumDiag}_{FN}$$ (2)

The total number of ‘avoidable deaths’ from all causes among Aboriginal and Torres Strait Islander cancer patients due to differences in relative survival (AvoidDth_All_FN_ (*t*)) was the difference between the predicted and expected number of all-cause deaths.


$${AvoidDth}_{All\_FN}(t)={PredDth}_{All\_FN}(t)-{ExpDth}_{All\_FN}(t)$$ (3)

The number of `avoidable cancer-related deaths’ (AvoidDth_Cancer_FN_ (*t*)) was calculated in a similar way, by substituting the all-cause probability of death estimates in Eqs (1) and (2) with the equivalent crude probability of cancer death estimates. A similar approach can be used to obtain the number of avoidable deaths from other causes based on crude probability of deaths from other causes. Both measures were also expressed as a percentage of the corresponding number of observed deaths. In addition, the proportion of total `avoidable’ deaths due to cancer (AvoidDth_Cancer_FN_ (*t*) /AvoidDth_All_FN_ (*t*)) was also calculated (expressed as a percentage).

The total number of avoidable deaths from all causes and cancer over the entire study period were also calculated.

We used the *standsurv* package to calculate the annual average number of avoidable total deaths for Aboriginal and Torres Strait Islander peoples diagnosed with cancer over the entire study period or last five years (2012–2016) from the model-based predicted standardised all-cause probability of death [20, 22]. Estimated crude probabilities of cancer death were used to calculate the number of avoidable cancer deaths [9]. The Stata codes for these calculations were adapted from previously published syntax for calculating avoidable deaths if colon cancer patients in the most socioeconomically deprived group had the same relative survival or crude probability of cancer death as the least deprived group by Syriopoulou and colleagues [20].

Statistical analyses were performed with Stata/SE version 16 (Stata, **RRID:SCR_012763,** StataCorp, TX, USA). Models were fitted with the *stpm2* package [21] and the *standsurv* package [23] used to generate all reported outcome measures. Associated 95% confidence intervals (CI) were estimated using delta method. Sample Stata syntax is provided in S1 Appendix.

#### Sensitivity analysis

Sensitivity analysis to assess the impact of unknown Aboriginal and Torres Strait Islander status on crude probability measure was carried out by repeating the analyses with an expanded cohort assuming that all these unknown cases were (i) other Australians; (ii) Aboriginal and Torres Strait Islander or (iii) equally and randomly distributed over both categories. The robustness of survival estimates to the choice of knots for flexible parametric models was also assessed.

## Results

**Fig 1:**
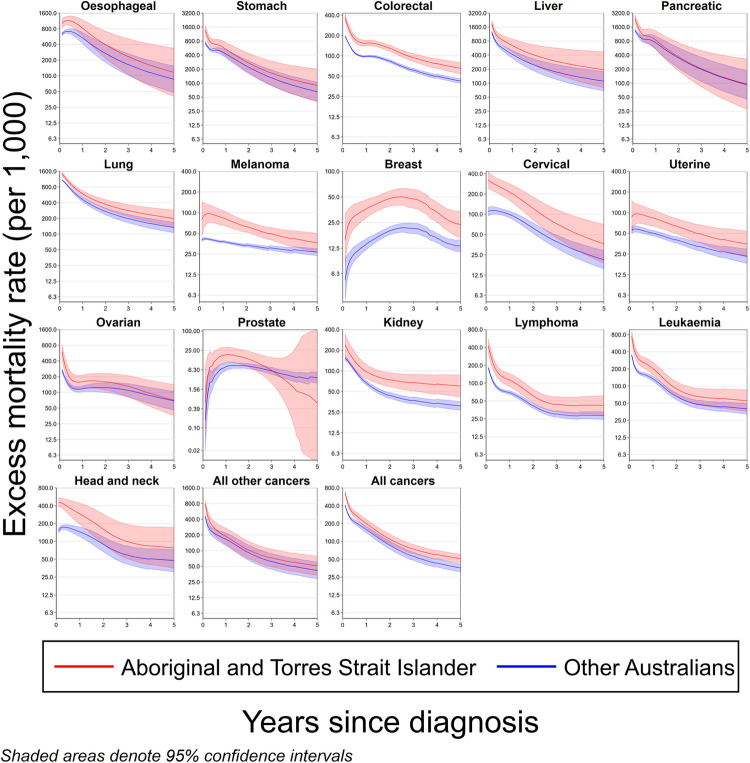
Excess mortality rate from all causes, up to five years since diagnosis by cancer type and Australian Indigenous status, Australia, 2005–2016.

Out of 709,239 cancer patients in the final study cohort, 12,830 (1.8%) identified as Aboriginal and Torres Strait Islanders (Table 1). For each cancer type, with the exception of prostate and pancreatic cancers, Aboriginal and Torres Strait Islander peoples had higher excess mortality during first five years of diagnosis than other Australians (Fig 1), with the magnitude of the effect varying by cancer type.

**Table 1:** Estimated standardised crude probability of death from cancer, death from other causes and being alive, Aboriginal and Torres Strait Islanders and disparity in estimated probabilities of death, to other Australians, Australia, five years since diagnosis, by cancer type, Australia, 2005–2016.

		Aboriginal and Torres Strait Islander		Aboriginal and Torres Strait Islander	
		5-year standardised crude probability^a^^,^^b^^,^^c^ [95% CI]		Disparity in standardised crude probability of death to other Australians (95% CI)^a^^,^^b^^,^^d^	
Cancer type	Cases (n)	Death from cancer	Death from other causes	Death from cancer	Death from other causes
Oesophageal (C15)	299	0.86 [0.82, 0.90]	0.03 [0.03, 0.04]	0.11 [0.07, 0.15]	0.01 [0.01, 0.02]
Stomach (C16)	290	0.76 [0.70, 0.81]	0.05 [0.04, 0.06]	0.09 [0.04, 0.14]	0.02 [0.01, 0.03]
Colorectal (C18-20)	1,255	0.38 [0.35, 0.41]	0.09 [0.09, 0.09]	0.10 [0.07, 0.13]	0.04 [0.04, 0.04]
Liver (C22)	449	0.88 [0.85, 0.91]	0.03 [0.02, 0.03]	0.13 [0.09, 0.16]	0.01 [0.00, 0.01]
Pancreatic (C25)	387	0.89 [0.85, 0.92]	0.02 [0.02, 0.03]	0.04 [0.00, 0.07]	0.01 [0.01, 0.01]
Lung (C33-34)	2,033	0.86 [0.84, 0.87]	0.03 [0.03, 0.03]	0.07 [0.05, 0.09]	0.01 [0.01, 0.02]
Melanoma (C43)	433	0.16 [0.13, 0.21]	0.10 [0.09, 0.10]	0.06 [0.02, 0.10]	0.04 [0.04, 0.05]
Female Breast (C50)	1,677	0.17 [0.15, 0.20]	0.07 [0.07, 0.07]	0.09 [0.06, 0.11]	0.04 [0.04, 0.04]
Cervical (C53)	343	0.44 [0.40, 0.50]	0.02 [0.02, 0.03]	0.20 [0.15, 0.25]	0.01 [0.01, 0.01]
Uterine (C54-C55)	476	0.18 [0.14, 0.24]	0.07 [0.07, 0.08]	0.04 [0.00, 0.09]	0.04 [0.04, 0.05]
Ovarian (C56)	179	0.51 [0.45, 0.59]	0.04 [0.03, 0.04]	0.10 [0.03, 0.17]	0.02 [0.01, 0.02]
Prostate (C61)	1,233	0.07 [0.05, 0.09]	0.16 [0.16, 0.17]	0.02 [0.00, 0.05]	0.08 [0.07, 0.08]
Kidney (C64)	307	0.28 [0.22, 0.34]	0.08 [0.07, 0.09]	0.08 [0.02, 0.14]	0.04 [0.04, 0.05]
Lymphoma (C81-86)	463	0.28 [0.24, 0.33]	0.07 [0.06, 0.07]	0.10 [0.06, 0.14]	0.03 [0.02, 0.03]
Leukaemia (C91-95)	367	0.46 [0.41, 0.52]	0.05 [0.05, 0.06]	0.13 [0.08, 0.19]	0.02 [0.01, 0.03]
Head and neck (C00-14, C30-32)	974	0.54 [0.51, 0.57]	0.06 [0.05, 0.06]	0.21 [0.18, 0.24]	0.02 [0.02, 0.03]
All other cancers (C17, C21, C23-C24, C26-C29, C35-C42, C44-C49, C51-C52, C57-C60, C62-C63, C65-C80, C87-C90, C96-C97, D45, D46, D47.1, D47.3-D47.5)	1,665	0.46 [0.44, 0.47]	0.05 [0.05, 0.06]	0.06 [0.04, 0.08]	0.03 [0.02, 0.03]
All cancers (C00-C97, D45, D46, D47.1, D47.3-D47.5)	12,830	0.44 [0.44, 0.45]	0.07 [0.07, 0.07]	0.07 [0.07, 0.08]	0.03 [0.03, 0.03]

ICD-10 International Statistical Classification of Diseases and related health problems, Tenth revision, CI Confidence intervals

^a.^ See Method section for details of calculations. All reported measures were standardised to the covariate distribution of the Aboriginal and Torres Strait Islander cancer cohort.

^b.^ Estimated using *standsurv* package

^c.^ Crude probability of being alive is 1-total crude probability of death

^d.^ Difference between the standardised crude probability of death from cancer or other causes for Aboriginal and Torres Strait Islander cancer patients and the corresponding standardised crude probability of death from cancer or other causes for other Australians.

### Crude probability of death among Aboriginal and Torres Strait Islanders

For all cancers combined, the standardised five-year crude probability of cancer death among Aboriginal and Torres Strait Islanders was 0.44 and other causes was 0.07. Values varied by cancer type with estimated probabilities of deaths from cancer being at least 0.86 for oesophageal, liver, lung, and pancreatic cancers, and lowest (0.07) for prostate cancer (Table 1). The corresponding probability of dying from other causes was highest (0.16) for prostate cancer while less than 0.04 for oesophageal, liver, lung, pancreatic and cervical cancers.

**Fig 2:**
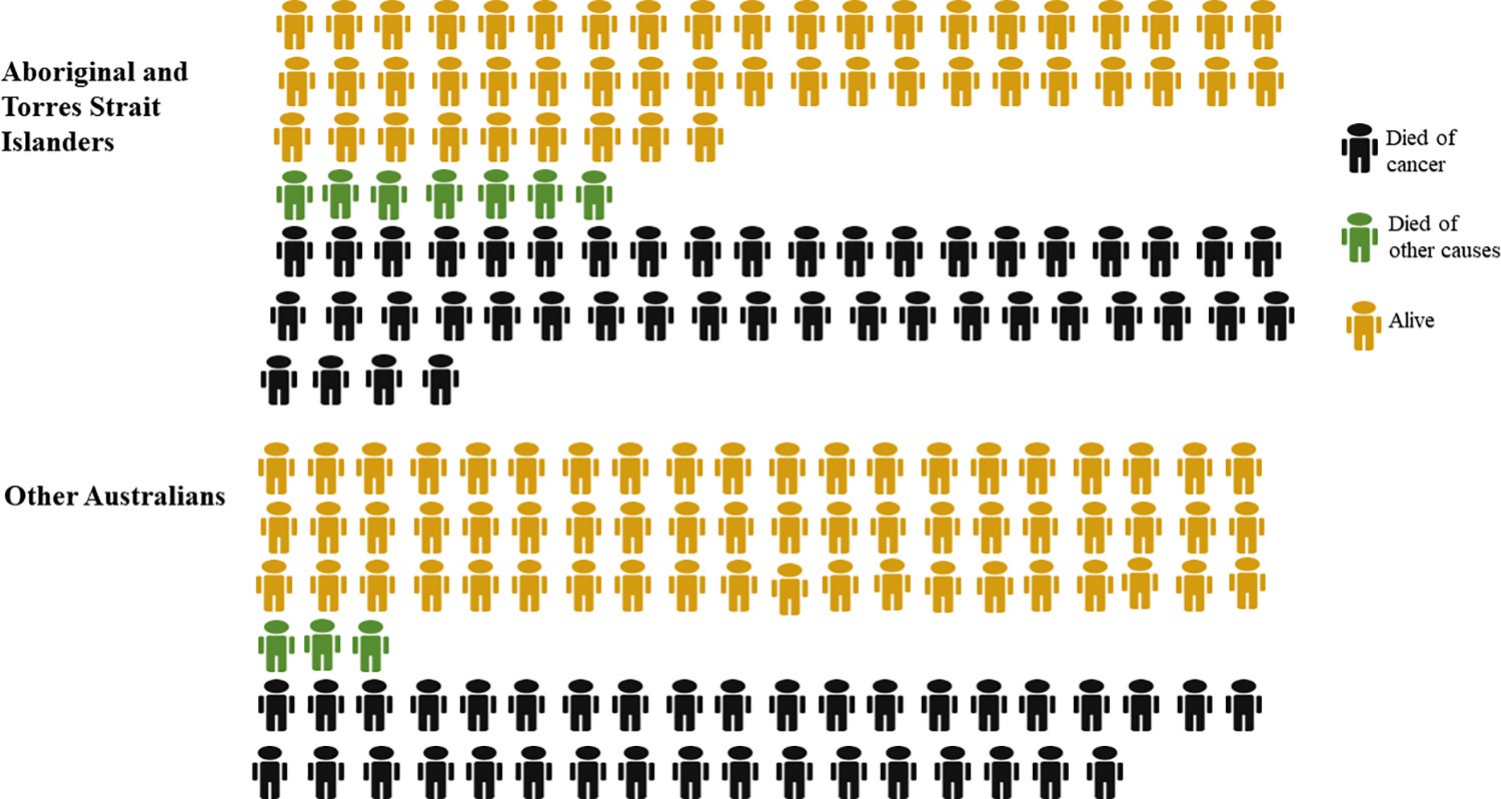
Five-year crude probability of death from cancer, death from other causes and being alive, for all cancers combined by Aboriginal and Torres Strait Islander status, Australia, 2005–2016.

Estimated crude probabilities can also be presented in terms of natural frequencies. For illustration, based on information in Table 1, among 100 Aboriginal and Torres Strait Islander people diagnosed with cancer, we would expect 44 deaths from cancer within five years of diagnosis, seven deaths from other causes and 49 to still be alive (Fig 2).

### Disparity in crude probability of death

**Fig 3:**
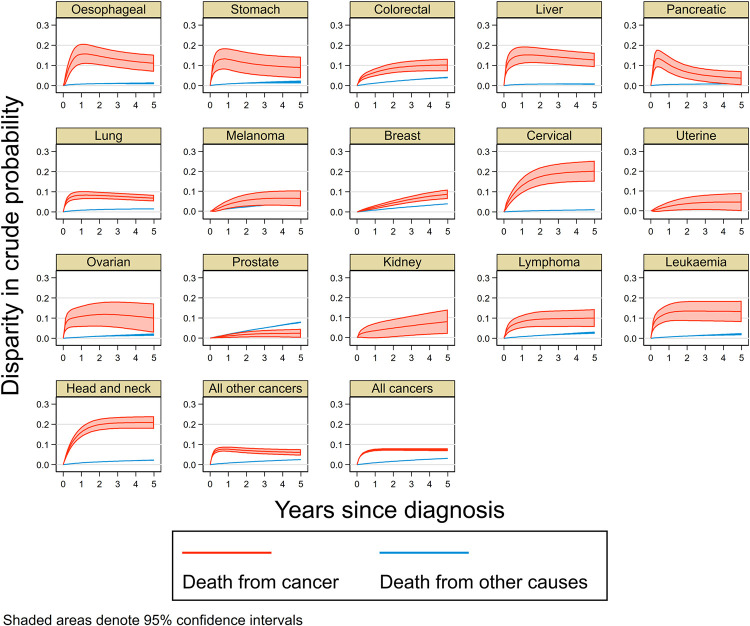
Disparity in standardised crude probability of death from cancer (a) and other causes (b), five years since diagnosis between Aboriginal and Torres Strait Islanders and other Australians, by cancer type, Australia, 2005–2016.

Estimated crude probabilities of dying from cancer and other causes within five years of diagnosis were consistently higher among Aboriginal and Torres Strait Islander peoples than other Australians, after standardising to the same covariate distribution as Aboriginal and Torres Strait Islanders for all included cancer types and groups (Table 1, Fig 3). For all cancers combined, these probabilities were 0.07 and 0.03 higher than among other Australians respectively. The magnitude of this differential varied by cancer type. For example, the disparity for cancer deaths was around 0.20 for cervical and head and neck cancers but less than 0.05 for pancreatic, prostate, and uterine cancers (Table 1, Fig 3). Disparities for other causes of death were generally lower, ranging from 0.01 for oesophageal, liver, lung, pancreatic and cervical cancers to 0.08 for prostate cancer.

### Crude probability of death and the disparity by time period: All cancers combined

Estimated crude probability of death from cancer and other causes for Aboriginal and Torres Strait Islanders between 2005–2010 for all cancers combined were 0.46 [95% CI 0.45, 0.47] and 0.07 [95% CI 0.07, 0.07] respectively. Corresponding values for 2011–2016 were 0.43 [95% CI 0.42, 0.45] and 0.07 [95% CI 0.06, 0.07]. For other Australians, these probabilities were 0.38 [95% CI 0.38, 0.39] and 0.03 [95% CI 0.03, 0.3] from 2005–2010 and 0.35 [95%CI 0.35, 0.36] and 0.04 [95% CI 0.04, 0.04] from 2011–2016. Hence the crude probability of cancer death for Aboriginal and Torres Strait Islander people was 0.08 higher than for other Australians over both time periods and around 0.04 higher for deaths from other causes.

### Avoidable deaths

We estimated that among a typical cohort of 1,269 Aboriginal and Torres Strait Islander peoples diagnosed per year between 2012–2016, about 133 of the 646 observed deaths from any cause within five years of diagnosis were potentially avoidable if relative survival was same as for other Australians, with 94 (71%) of these being due to cancer (Table 2, S1 Fig). Estimates varied by cancer type with around 21 avoidable deaths for head and neck and female breast cancer, whereas not more than three deaths were avoidable for ovarian, pancreatic and stomach cancers. For cervical, ovarian, liver, pancreatic, and head and neck cancers, at least 90% of estimated avoidable deaths were due to cancer by contrast to a quarter for prostate cancer.

**Table 2:** Average annual number and percentage of avoidable deaths from all-causes and cancer and percentage of avoidable all-cause deaths due to cancer if Aboriginal and Torres Strait Islander cancer patients had the same survival as other Australians, Australia, 2012–2016.

	Aboriginal and Torres Strait Islander									
		Death from cancer and other causes				Death from cancer				
		Number of deaths		Avoidable deaths		Number of deaths		Avoidable deaths		% Avoidable deaths from cancer
Cancer type (ICD-10)	Average annual Cases (C_ATSI_)^a^	Observed^b^^,^^c^ (OD_ALL_)	Expected^b^^,^^d^ (ED_ALL_)	Number^b^ (AD_ALL_)_:_ OD_ALL_—ED_ALL_	% Observed ((AD_ALL_/OD_ALL_) *100)	Observed^b^^,^^e^(OD_CANCER_)	Expected^b^^,^^f^ (ED_CANCER_)	Number^b^ (AD_CANCER_): OD_CANCER_—ED_CANCER_	% Observed ((AD_CANCER_ /OD_CANCER_) *100)	((AD_CANCER_/A_ALL_) *100)
Oesophageal	29	26 [25, 27]	22 [22, 23]	4 [2, 5]	14	25 [24, 26]	22 [21, 22]	3 [2, 4]	13	75
Stomach	27	22 [21, 23]	19 [18, 19]	3 [2, 4]	13	20 [19, 22]	18 [18, 18]	2 [1, 4]	12	67
Colorectal	118	55 [52, 59]	38 [38, 39]	17 [14, 20]	31	45 [41, 48]	33 [32, 33]	12 [8, 16]	27	71
Liver	48	44 [42, 45]	37 [36, 38]	6 [5, 8]	15	42 [41, 44]	36 [35, 37]	6 [4, 8]	14	100
Pancreatic	42	38 [37, 40]	36 [36, 37]	2 [1, 3]	5	37 [36, 39]	36 [35, 36]	2 [0, 3]	4	100
Lung	202	180 [177, 183]	163 [162, 164]	17 [14, 20]	9	172 [169, 176]	159 [158, 160]	14 [11, 17]	8	82
Melanoma	41	11 [9, 12]	6 [6, 6]	4 [3, 6]	42	7 [5, 9]	4 [4, 4]	3 [1, 4]	39	75
Female Breast	172	42 [38, 46]	20 [20, 20]	22 [18, 26]	52	29 [26, 34]	14 [14, 15]	15 [11, 19]	51	68
Cervical	30	14 [13, 16]	8 [7, 8]	6 [5, 8]	45	13 [12, 15]	7 [7, 8]	6 [4, 8]	45	100
Uterine	46	12 [10, 14]	8 [7, 8]	4 [2, 6]	34	9 [7, 11]	6 [6, 7]	2 [–0, 4]	24	50
Ovarian	15	8 [7, 8]	6 [6, 7]	2 [1, 3]	21	8 [7, 9]	6 [6, 6]	2 [0, 3]	19	100
Prostate	117	26 [24, 29]	15 [14, 15]	12 [10, 14]	45	8 [5, 11]	5 [5, 5]	3 [0, 5]	36	25
Kidney	37	13 [11, 15]	9 [8, 9]	4 [2, 7]	34	10 [8, 13]	7 [7, 7]	3 [1, 5]	29	75
Lymphoma	48	17 [15, 19]	11 [10, 11]	6 [4, 8]	37	14 [12, 16]	9 [9, 9]	5 [3, 7]	35	83
Leukaemia	39	20 [18, 22]	14 [14, 14]	6 [4, 8]	30	18 [16, 20]	13 [12, 13]	5 [3, 7]	29	83
Head and neck	90	53 [51, 56]	33 [32, 34]	21 [18, 23]	39	48 [46, 51]	30 [29, 31]	19 [16, 22]	39	90
All other cancers	154	79 [77, 81]	66 [65, 66]	13 [11, 15]	17	71 [68, 73]	61 [61, 62]	10 [7, 12]	13	69
All cancers	1,269	646 [639, 654]	513 [511, 515]	133 [125, 141]	21	563 [555, 572]	470 [468, 472]	94 [85, 102]	17	71

ICD-10 International Statistical Classification of Diseases and related health problems, Tenth revision, CI Confidence intervals

^a.^ The average annual number of cases among Aboriginal and Torres Strait Islanders, 2012–2016

^b.^ Estimated using *standsurv* package

^c.^ Product of the number of cases and the standardised probability of all-cause death for Aboriginal and Torres Strait Islanders

^d.^ Product of the number of cases and the standardised probability of all-cause death for Aboriginal and Torres Strait Islanders if they had the same cancer mortality (relative survival) as other Australians.

^e.^ Product of the number of cases and the standardised crude probability of cancer death for Aboriginal and Torres Strait Islanders

^f.^ Product of the number of cases and the standardised probability of cancer death for Aboriginal and Torres Strait Islanders if they had the same cancer mortality (relative survival) as other Australians.

The total number of avoided deaths over the entire study period (2005–2016) if Aboriginal and Torres Strait Islander peoples had the same relative survival as other Australians was 1,348, with 947 of these deaths due to cancer. Estimates varied by cancer type with around 128 avoided deaths for colorectal, 139 for lung, 147 for female breast and 203 for head and neck cancers. Less than 20 deaths were avoided for pancreatic and ovarian cancers.

The number of avoidable deaths from cancer increased sharply over the first few months after cancer diagnosis before plateauing (S2 Fig). By contrast, the corresponding estimates for other cause deaths increased consistently over the time following cancer diagnosis. For all cancers combined, 65% of avoidable all-cause deaths occurred within first six months after diagnosis, 78% in the first year and 88% within the first two years after diagnosis. While patterns varied somewhat by cancer type, in general there was a rapid accumulation of avoidable deaths within the first six months after diagnosis except for prostate, female breast and uterine cancers (S3 Fig).

### Sensitivity analysis

Estimates were relatively robust to the number (and position of knots) used for the modelling, with for example the crude probability of cancer death for all cancers combined ranging from 0.44 to 0.46 for Aboriginal and Torres Strait Islander peoples depending on the specific selection., The corresponding disparity was around 0.07 in each instance. Sensitivity analysis for unknown Aboriginal and Torres Strait Islander status (S1 Table) showed that although the magnitude of the estimates varied, the overall patterns were similar. For example, the crude probability of cancer deaths for all cancers combined among Aboriginal and Torres Strait Islanders ranged from 0.36 to 0.44 depending on the assumption made, while the corresponding disparity varied from 0.04 to 0.08.

## Discussion

This study, a first for Australia, highlighted how Aboriginal and Torres Strait Islander peoples diagnosed with any type of cancer have a consistently higher probability of dying from cancer or from other causes within five years of diagnosis compared to other Australians. The effect of this disparity was observed in avoidable loss of life; nearly one-fifth of the observed 646 deaths among Aboriginal and Torres Strait Islander peoples within five years of their cancer diagnosis would have been avoided if there were no survival disparities, with the majority of these avoidable deaths being caused by cancer.

A key advantage of these analyses is the ability to separate out the contribution of cancer causes and other causes to the estimated number of avoidable deaths, something previous studies have not done. For example, in Queensland it was estimated that around 19% of cancer deaths were avoidable among Aboriginal and Torres Strait Islander cancer patients from 2008–2012, but by using cause-specific survival it did not incorporate the impact of other causes of death [24].

Underlying reasons for the survival disparities after a cancer diagnosis are multifaceted and in practice may be difficult to eliminate. Although beyond the scope of this study, it has been suggested that key contributors likely include diagnostic, clinical, access-related, behavioural, and environmental factors [4, 25–32]. Previous studies have shown that Aboriginal and Torres Strait Islander peoples are more likely to be diagnosed with advanced disease [4, 27, 33–35]. They also have lower rates of participation in Australia’s population-based national breast bowel and cervical cancer screening programs than other Australians [1]. Hence it is likely that at least some of the observed disparities reported in this study are due to differences in stage at diagnosis. This is even more so given the disparity in the crude probability of cancer death typically peaked in the first year after cancer diagnosis. Unfortunately, as is the case with most population-based cancer registries, stage information is not routinely collected in Australian Cancer Registries, thus limiting our ability to understand how much of the survival disparity is due to variations in stage at diagnosis [36, 37] compared to other factors such as treatment.

Several studies have also shown that reducing the number of comorbidities and increasing treatment utilisation could reduce the survival disparities between Aboriginal and Torres Strait Islander peoples and other Australians diagnosed with breast [27, 30], liver [32], lung [25] or cervical [33] cancer.

In addition, disparities in the social determinants of health such as social, geographical and economic disadvantage for Aboriginal and Torres Strait Islanders are well recognised as influencing cancer outcomes for some cancer types [26, 38]. For example, previous studies have shown that adjusting for socio-economic status markedly attenuated the disparity for female breast cancer [30], however this effect was not evident for head and neck cancers [29] or all cancers combined [3].

Additional research using the latest available data to investigate the overlap and causal links between factors associated with survival experience of Aboriginal and Torres Strait Islander peoples following a cancer diagnosis is crucial to provide a broader and more comprehensive understanding of the main drivers of existing dipartites, and where interventions are best targeted to reduce this gap. Reducing the burden of cancer will require focussed and coordinated actions addressing a range of issues including prevention and early detection, optimal treatment, and care that is responsive to the needs of Aboriginal and Torres Strait Islander people [39, 40].

The crude probability of death and avoidable deaths estimates are highly dependent on the time since diagnosis, and ultimately, if we followed up all patients for a sufficient number of years there would be no deaths avoided. In first few years since diagnosis, cancer mortality is the largest contributor to prognostic outcomes [7, 9]. At five years post diagnosis, there is evidence that the number of avoidable cancer deaths is starting to plateau, while the number of avoidable deaths due to other causes consistently increases. This is consistent with the observation that mortality from other causes becomes increasingly important with increasing follow-up time, in part because of increasing age of the prevalent cases [41]. This, combined with the cancer survival disparity between Aboriginal and Torres Strait Islanders and other Australians being greatest in the first few years after diagnosis [2, 42], for studies having longer follow up intervals, or using measures such as remaining life expectancy [41], the impact of non-cancer causes of death on the disparity between Aboriginal and Torres Strait Islander and other Australian cancer patients will likely be higher.

Study strengths include use of high-quality population-based cancer registry data [15] from four Australian/states and territories with high quality Aboriginal and Torres Strait Islander information. Flexible parametric relative survival models allowed partitioning of all-cause probability of death into cancer and other causes without requiring cause of death information [8] and estimation of these probabilities standardised to the covariate distribution of Aboriginal and Torres Strait cancer cohort thereby allowing a fair comparison between two population groups [20]. Moreover, we quantified the population-level impact of survival disparities through avoidable deaths, an absolute measure which incorporates the impact of other causes of death, the average number of cases and the survival differences between the two cohorts by Aboriginal and Torres Strait Islander status.

While there was no evidence that the Aboriginal and Torres Strait Islander disparity in crude probabilities of death from cancer or other causes changed over time for all cancers combined, we were not able estimate this for individual cancer types due to the lack of statistical convergence.

We also focused our analysis on those four cancer registries with high quality information on Aboriginal and Torres Strait Islander status [1, 14], covering 84% of Australia’s Aboriginal and Torres Strait Islander population. These registries combine data on self-reported status from multiple datasets and apply algorithms to derive the most plausible status [3, 43]. It is not known to what extent our results can be extrapolated to the remaining jurisdictions of Australia. Finally, although we needed to exclude people with unknown Aboriginal and Torres Strait Islander status (6% of cohort) from the main analyses, the magnitude, and general patterns of the disparities in the reported measures were robust to various assumptions made about their true distribution.

## Conclusions

Aboriginal and Torres Strait Islander peoples diagnosed with cancer have higher probability of dying from cancer within first five years of diagnosis across a range of cancer types even after accounting for competing mortality than other Australians. By splitting the deaths from all causes into deaths due to cancer and deaths due to other causes this study provided a unique perspective into the impact of survival disparities and further understanding of the excess deaths among Aboriginal and Torres Strait Islander cancer patients. Interventions designed to improve early diagnosis and management, increase screening participation, and reduce risk factor prevalence especially smoking among Aboriginal and Torres Strait Islander peoples could lead to more equitable outcomes for multiple cancer types in particular female breast, colorectal, lung, cervical and head and neck cancers within first five years after diagnosis.

## Supporting information

S1 Appendix Example Stata syntax. (PDF)

S1 Table Sensitivity analysis for unknown Aboriginal and Torres Strait Islander status. Estimated standardised crude probabilities of death and being alive, five years since diagnosis, from the sensitivity analysis assuming various distributions for true Aboriginal and Torres Strait Islander status by selected cancer type Australia, 2005–2016.
(PDF)

S1 Fig Average annual number of ‘avoidable’ cancer and other cause deaths, 5 years after diagnosis, by cancer type, Aboriginal and Torres Strait Islanders, Australia, 2005–2016. (TIF)

S2 Fig Average annual number of ‘avoidable’ cancer and other cause deaths, all cancers combined, up to 5 years since diagnosis, Aboriginal and Torres Strait Islanders, Australia, 2012–2016. (TIF)

S3 Fig Average annual number of ‘avoidable’ cancer and other cause deaths, all cancers combined, up to 5 years since diagnosis, Aboriginal and Torres Strait Islanders, Australia, 2012–2016. (TIF)
