# Supplementary material for: Quantifying the number of deaths among Aboriginal and Torres Strait Islander cancer patients that could be avoided by removing survival inequalities, Australia 2005–2016
Source: PLoS One. 2022 Aug 26;17(8):e0273244. doi: 10.1371/journal.pone.0273244 (PMC9417002; doi:10.1371/journal.pone.0273244)
Supplement: S1 Appendix — (PDF) [file pone.0273244.s001.pdf]

S1 Appendix Example Stata syntax.

This Appendix provides the Stata code used to estimate reported outcome measures for this paper. Flexible parametric relative survival models were fitted using *stpm2*. All predictions were obtained using command *standsurv*.

You can install these packages within Stata using

```
ssc install stpm2
ssc install standsurv
```

The population mortality file (popmort.dta) contains the variables state (state, 1=NSW, 3=QLD, 5=WA, 7=NT), year of death (_year, 2005-2016), sex (sex, 1=Male, 2=Female), Aboriginal and Torres Strait Islander status (ind, 1=other, 2=Aboriginal and Torres Strait Islander) and age at death (_age, 0-84). It is sorted by variables state, year of death, sex, Aboriginal and Torres Strait Islander status and age at death.

As an illustrative example we use dataset for colorectal cancer which contains the following variables: ID number (id), state (state, 1=NSW, 3=QLD, 5=WA, 7=NT), age at diagnosis in years (dxage, 15-84), year of diagnosis (yeardx, 2005-2016), date of diagnosis in days (assumed to be the 15th of each month), month and year (dxdate), sex (sex, 0 = Male, 1 = Female), survival time in days till 31 December 2016 (days between), survival status (death, 0 = Alive, 1 = Dead) and Aboriginal and Torres Strait Islander status (ind, 1=other Australians, 2=Aboriginal and Torres Strait Islander).

```
/* declare data to be survival data */
use colorectal, clear
stset daysbetween, id(id) scale(365.25) fail(death=1)

/* Match with population mortality rate */
gen _age = min(int(dxage + _t),84)
gen _year = int(yeardx + _t)
sort state _year sex ind _age
merge m:1 state _year sex ind _age using _popmort, keep(match master)
keepusing(rate)
drop _age _year _merge

/* Use splines for age, df=4 */
rcsgen dxage, gen(sage) df(4) orthog

/* Generate dummy variables for state, being Aboriginal and Torres Strait
Islander and being female as standsurv does not recognise factor variables
*/
tab state, gen(state)
gen indig = ind== 1
gen fem = sex==2

/* Fit the flexible parametric model - this is the model for a specific
cancer type. Model for all cancers combined, all other cancers or head and
neck cancers were also adjusted for broad cancer type and interactions
between broad cancer type and sex or age */
```

```
stpm2 indig sage* fem state2-state4, scale(haz) df(4) bhazard(rate) nolog
tvc(sage* fem state2-state4 indig) dftvc(2)

/* Create a temporary time variable used for predictions */
range tt 0 5 6
```

Probability of death from all-causes

We use the *standsurv* command for generating probabilities of death from all-causes by specifying the *failure* option. The *if* statement is used to set the covariate distribution to that for Aboriginal and Torres Strait Islanders.

```
/* Generating the standardised probability of death from all causes */

standsurv if indig ==1, timevar(tt) ///
failure /// Probabilities of death from all-causes
ci /// Obtain confidence intervals
atvar(crpr1 crpr2)  /// Name of new variables
at1(indig 0) /// Prediction for other Australians
at2(indig 1) /// Prediction for Aboriginal and Torres Strait Islanders
contrast(difference) /// Calculate the difference between groups
contrastvar(diff_ind) /// New variables containing the difference
expsurv(using (_popmort) /// Popmort file
datediag(dxdate) /// Date of diagnosis in the dataset
agediag(dxage) /// Age at diagnosis in the dataset
pmrate(rate) /// Rate variable in popmort file
pmage(_age) /// Age variable in popmort file
pmyear(_year) /// Year variable in popmort file
pmother(sex state ind ) /// Other variables in popmort file
pmmaxyear(2016) /// Maximum year in popmort file
pmmaxage(84) ///  Maximum age in popmort file
at1(ind 0) /// Expected rate for other Australians
at2(ind 1) /// Expected rate for Aboriginal and Torres Strait Islanders
)
```

Crude probabilities of death

We use the *standsurv* command for generating crude probabilities of death from cancer and other causes by specifying the *crudeprob* option.

```
/* Generating standardized crude probability of death from cancer and other
causes /*

standsurv if indig ==1, timevar(tt) ///
crudeprob /// Crude probabilities of death
ci /// Obtain confidence intervals
atvar(crpr1 crpr2)  /// Name of new variables
at1(indig 0) /// Prediction for other Australians
at2(indig 1) /// Prediction for Aboriginal and Torres Strait Islanders
contrast(difference) /// Calculate the difference between groups
contrastvar(diff_ind) /// New variables containing the difference
expsurv(using (_popmort) /// Popmort file
datediag(dxdate) /// Date of diagnosis in the dataset
agediag(dxage) /// Age at diagnosis in the dataset
pmrate(rate) /// Rate variable in popmort file
pmage(_age) /// Age variable in popmort file
```

```
pmyear(_year) /// Year variable in popmort file
pmother(sex state ind ) /// Other variables in popmort file
pmmaxyear(2016) /// Maximum year in popmort file
pmmaxage(84) ///  Maximum age in popmort file
at1(ind 0) /// Expected rate for other Australians
at2(ind 1) /// Expected rate for Aboriginal and Torres Strait Islanders
)
```

Avoidable deaths from all-causes

We want to use the *standsurv* command to estimate the number of avoidable deaths from all-causes among Aboriginal and Torres Strait Islanders if Aboriginal and Torres Strait Islanders had the same relative survival as other Australians. The covariate distribution is set to that for Aboriginal and Torres Strait Islanders.

```
/// Define counts that is the average number of cases diagnosed from 2012-
2016
count if yeardiag>=2012
local N AD = `r(N)'/5

/* Generating the number of avoidable deaths from all causes.
This is the difference between observed and expected number of deaths,
obtained by multiplying corresponding predicted all-cause probability of
death by the count*/

standsurv, timevar(tt) ///
failure /// Probabilities of death from all-causes
ci /// Obtain confidence intervals
atvar(dths_expt dths_obs)  /// Name of new variables
at1(indig 0, atif(indig==1)) /// Same relative survival Other Australians
at2(indig 1, atif(indig ==1) /// Aboriginal and Torres Strait Islanders
per('N AD') /// Multiplies predictions by the count
contrast(difference) /// Calculate the difference between groups
contrastvar(Adt) /// New variable for the difference
expsurv(using (_popmort) /// Popmort file
datediag(dxdate) /// Date of diagnosis in the dataset
agediag(dxage) /// Age at diagnosis in the dataset
pmrate(rate) /// Rate variable in popmort file
pmage(_age) /// Age variable in popmort file
pmyear(_year) /// Year variable in popmort file
pmother(sex state ind ) /// Other variables in popmort file
pmmaxyear(2016) /// Maximum year in popmort file
pmmaxage(84) ///  Maximum age in popmort file
at1(ind 1) /// Expected rate for Aboriginal and Torres Strait Islanders
at2(ind 1) /// Expected rate for Aboriginal and Torres Strait Islanders
)
```

Avoidable deaths from cancer

We want to use the *standsurv* command to estimate the number of avoidable deaths from cancer or other causes among Aboriginal and Torres Strait Islanders if Aboriginal and Torres Strait Islanders had the same relative survival as other Australians. The covariate distribution is set to that for Aboriginal and Torres Strait Islanders.

```
/// Define counts that is the average number of cases diagnosed from 2012-
2016
count if yeardiag>=2012
local N AD = `r(N)'/5

/* Generating the number of avoidable deaths from cancer or other causes *
This is the difference between observed and expected number of deaths,
obtained by multiplying corresponding predicted crude probability of death
by the count*/

standsurv, timevar(tt) ///
crudeprob /// Crude Probabilities of death
ci /// Obtain confidence intervals
atvar(dths_expt dths_obs)  /// Name of new variables
at1(indig 0, atif(indig==1)) /// Same relative survival Other Australians
at2(indig 1, atif(indig ==1) /// Aboriginal and Torres Strait Islanders
per(‘N AD’) /// Multiplies predictions by the count
contrast(difference) /// Calculate the difference between groups
contrastvar(AD) /// New variable for the difference
expsurv(using (_popmort) /// Popmort file
datediag(dxdate) /// Date of diagnosis in the dataset
agediag(dxage) /// Age at diagnosis in the dataset
pmrate(rate) /// Rate variable in popmort file
pmage(_age) /// Age variable in popmort file
pmyear(_year) /// Year variable in popmort file
pmother(sex state ind ) /// Other variables in popmort file
pmmaxyear(2016) /// Maximum year in popmort file
pmmaxage(84) ///  Maximum age in popmort file
at1(ind 1) /// Expected rate for Aboriginal and Torres Strait Islanders
at2(ind 1) /// Expected rate for Aboriginal and Torres Strait Islanders
```

The total number of avoidable deaths from cancer or other causes among Aboriginal and Torres Strait Islanders if Aboriginal and Torres Strait Islanders had the same relative survival as other Australians was calculated for the entire study period by replacing the count to be the total number of cases diagnosed over the 12 years (2005-2016).
