# Supplementary material for: Quantifying the number of deaths among Aboriginal and Torres Strait Islander cancer patients that could be avoided by removing survival inequalities, Australia 2005–2016
Source: PLoS One. 2022 Aug 26;17(8):e0273244. doi: 10.1371/journal.pone.0273244 (PMC9417002; doi:10.1371/journal.pone.0273244)
Supplement: S1 Table — Estimated standardised crude probabilities of death and being alive, five years since diagnosis, from the sensitivity analysis assuming various distributions for true Aboriginal and Torres Strait Islander status by selected cancer type Australia, 2005–2016. (PDF) [file pone.0273244.s002.pdf]

S1 Table Sensitivity analysis for unknown Aboriginal and Torres Strait Islander status. Estimated standardised crude probabilities of death and being alive, five years since diagnosis, from the sensitivity analysis assuming various distributions for true Aboriginal and Torres Strait Islander status by selected cancer type Australia, 2005-2016

		**Aboriginal and Torres Strait Islander**		**Other Australians**	
		5-year standardised crude probability (%)[1,2,3] [95% CI]		5-year standardised crude probability (%)[1,2,3] [95% CI]	
**Cancer type**	**Unknown Aboriginal and Torres Strait Islander cases assumed to be**	**Death from cancer**	**Death from other causes**	**Death from cancer**	**Death from other causes**
Liver	Other Australians	0.88 [0.85, 0.91]	0.03 [0.02, 0.03]	0.75 [0.73, 0.77]	0.02 [0.02, 0.02]
	Aboriginal and Torres Strait Islander	0.80 [0.77, 0.82]	0.04 [0.03, 0.04]	0.76 [0.75, 0.78]	0.02 [0.02, 0.02]
	Random & equally distributed	0.83 [0.79, 0.87]	0.03 [0.03, 0.04]	0.76 [0.74, 0.77]	0.02 [0.02, 0.02]
	Excluded	0.88 [0.85, 0.91]	0.03 [0.02, 0.03]	0.75 [0.74, 0.77]	0.02 [0.02, 0.02]
Breast	Other Australians	0.17 [0.15, 0.20]	0.07 [0.07, 0.07]	0.08 [0.08, 0.08]	0.03 [0.03, 0.03]
	Aboriginal and Torres Strait Islander	0.13 [0.12, 0.14]	0.10 [0.10, 0.10]	0.09 [0.09, 0.09]	0.04 [0.04, 0.04]
	Random & equally distributed	0.16 [0.15, 0.17]	0.09 [0.09, 0.09]	0.09 [0.09, 0.09]	0.04 [0.04, 0.04]
	Excluded	0.17 [0.15, 0.20]	0.07 [0.07, 0.07]	0.08 [0.08, 0.09]	0.03 [0.03, 0.03]
Head and neck	Other Australians	0.53 [0.51, 0.56]	0.06 [0.05, 0.06]	0.32 [0.31, 0.33]	0.03 [0.03, 0.03]
	Aboriginal and Torres Strait Islander	0.46 [0.44, 0.47]	0.10 [0.10, 0.10]	0.34 [0.33, 0.35]	0.05 [0.05, 0.05]
	Random & equally distributed	0.44 [0.42, 0.46]	0.09 [0.09, 0.09]	0.35 [0.33, 0.36]	0.04 [0.04, 0.04]
	Excluded	0.54 [0.51, 0.57]	0.06 [0.05, 0.06]	0.33 [0.32, 0.34]	0.03 [0.03, 0.03]
All cancers	Oher Australians	0.43 [0.42, 0.43]	0.07 [0.07, 0.07]	0.34 [0.34, 0.34]	0.04 [0.04, 0.04]
	Aboriginal and Torres Strait Islander	0.38 [0.38, 0.39]	0.12 [0.12, 0.12]	0.30 [0.30, 0.30]	0.06 [0.06, 0.06]
	Random & equally distributed	0.36 [0.35, 0.36]	0.11 [0.11, 0.11]	0.32 [0.32, 0.32]	0.05 [0.05, 0.05]
	Excluded	0.44 [0.44, 0.45]	0.07 [0.07, 0.07]	0.37 [0.37, 0.37]	0.03 [0.03, 0.03]

CI 95% Confidence interval

1. See Method section for details of calculations. All reported measures were standardised to the covariate distribution of the Aboriginal and Torres Strait Islander cancer cohort
2. Estimated using *standsurv* package
3. Crude probability of being alive is 1-total crude probability of death
