# Supplementary figures and images for: Quantifying the number of deaths among Aboriginal and Torres Strait Islander cancer patients that could be avoided by removing survival inequalities, Australia 2005–2016
Source: PLoS One. 2022 Aug 26;17(8):e0273244. doi: 10.1371/journal.pone.0273244 (PMC9417002; doi:10.1371/journal.pone.0273244)

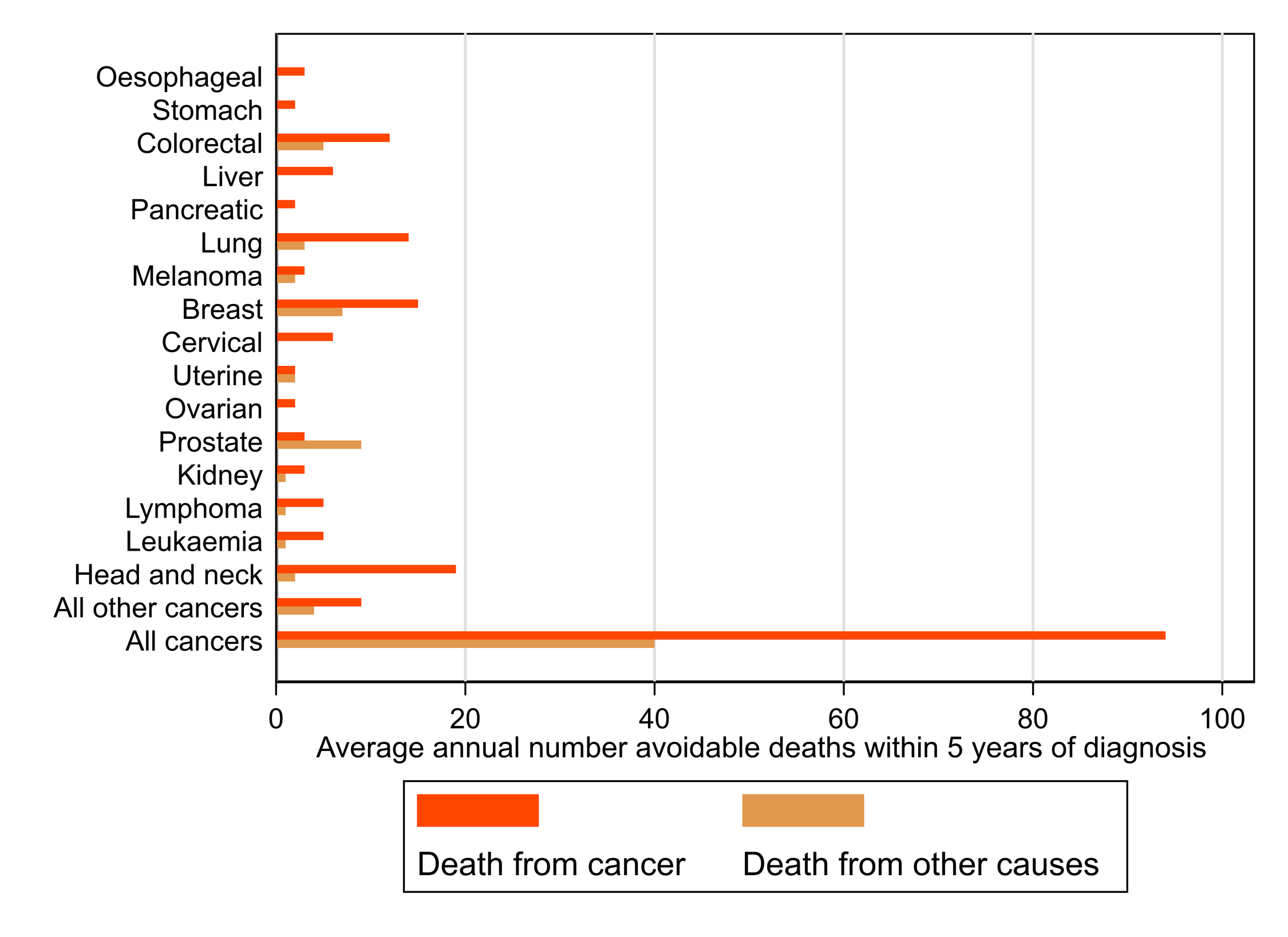

Supplement: S1 Fig — (TIF) [file pone.0273244.s003.tif]

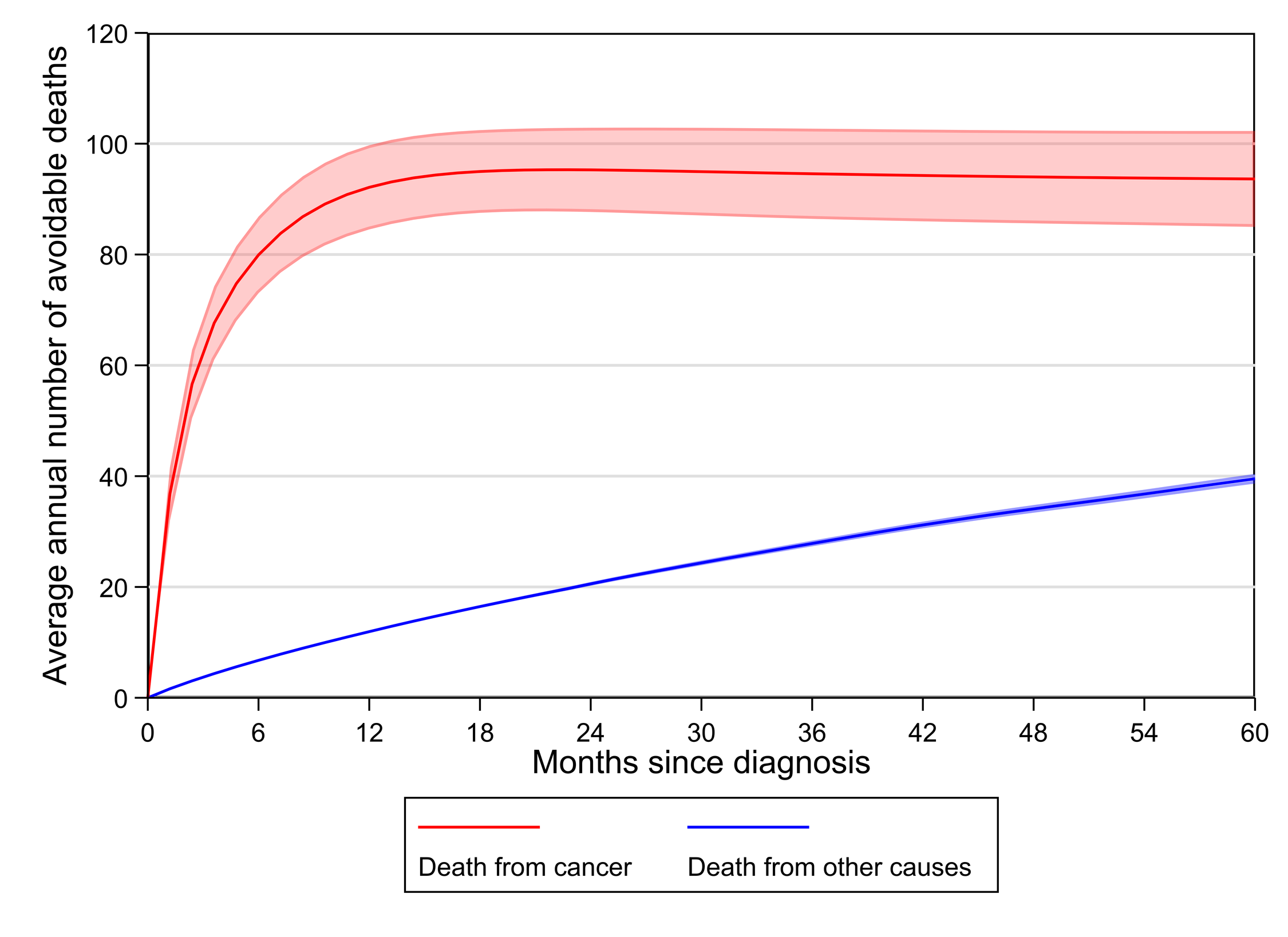

Supplement: S2 Fig — (TIF) [file pone.0273244.s004.tif]

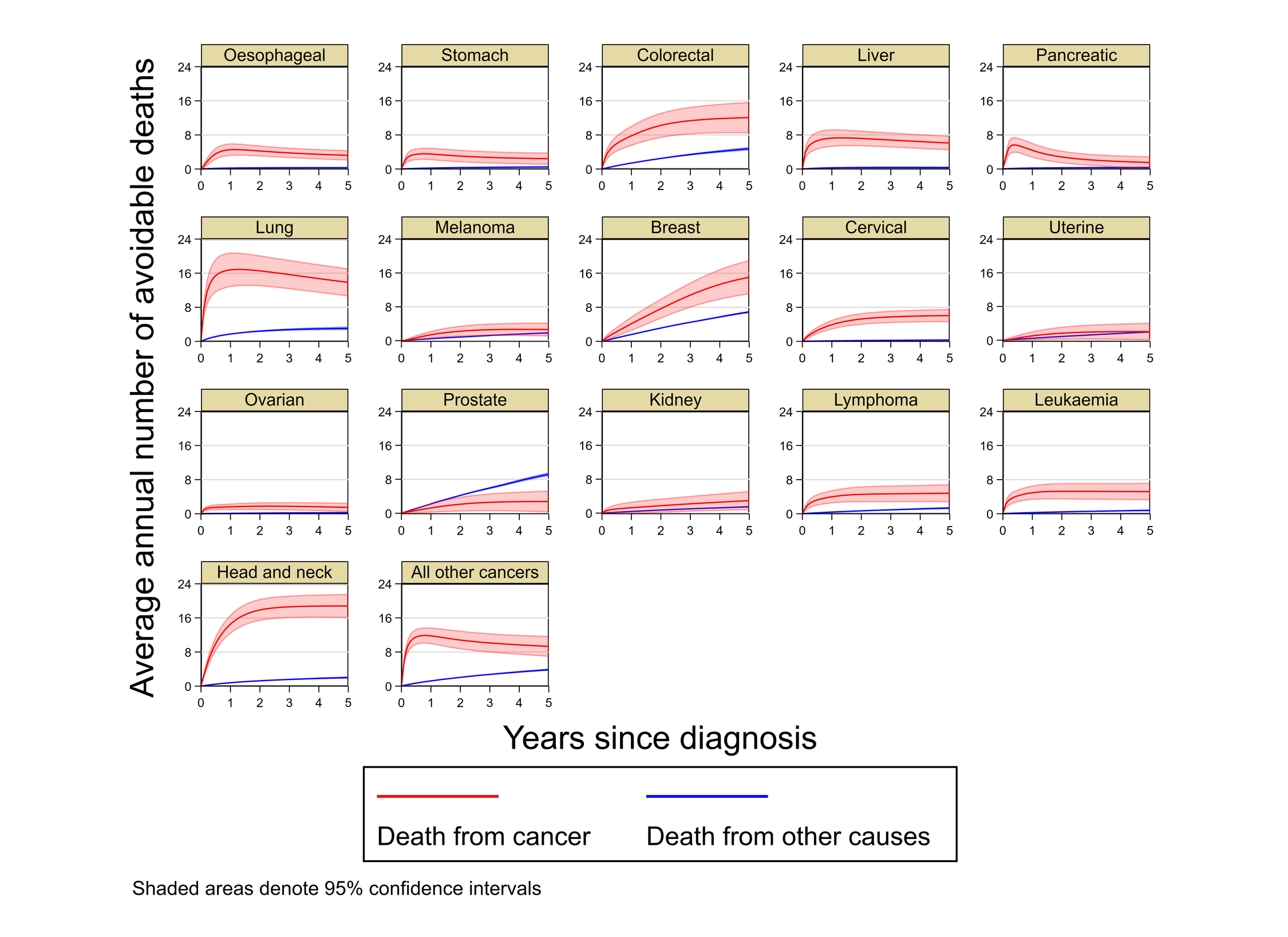

Supplement: S3 Fig — (TIF) [file pone.0273244.s005.tif]
